# Pulmonary embolism incidence among patient admitted under psychiatry department: a case-control study

**DOI:** 10.3389/fpsyt.2024.1449963

**Published:** 2024-08-16

**Authors:** Wanling Zhang, Dhirendra Paudel, Rui Shi, Junwei Yang, Jingwen Liu, Yanbin Jia

**Affiliations:** ^1^ Department of Psychiatry, First Affiliated Hospital of Jinan University, Guangzhou, Guangdong, China; ^2^ Department of Psychiatry, Sleep Medicine Center, Nanfang Hospital, Southern Medical University, Guangzhou, Guangdong, China; ^3^ Chongqing Mental Health Center, Chongqing, China; ^4^ Shenzhen Mental Health Center, Shenzhen Kangning Hospital, Shenzhen, China

**Keywords:** pulmonary embolism, deep vein thrombosis, venous thromboembolism, d-dimer, psychotropic drugs, mental disorders, computed tomography angiography

## Abstract

**Background:**

Pulmonary embolism (PE) is a serious and potentially life-threatening condition that requires prompt diagnosis and treatment. Identifying risk factors and diagnostic markers can aid in the early detection and management of this condition.

**Methods:**

This case-control study examined 10,077 patients admitted to Shenzhen Kangning Hospital’s psychiatry facility in 2020. Among these, 65 patients were diagnosed with PE, including 50 new cases. After survival sampling for controls and age-and-gender matching, the study included 41 new PE cases and 41 age-and-gender-matched controls. Data on demographics, comorbidities, and medication use were extracted from electronic records. Conditional logistic regression analyses were performed to determine the association between each predictor and PE risk. Additionally, the sensitivity and specificity of the d-dimer diagnostic tool were assessed.

**Results:**

In univariable conditional logistic regression, active alcoholism was associated with a higher PE risk (OR=3.675, 95% CI 1.02–13.14, P=0.046). A history of physical restraint (OR=4.33, 95% CI 1.24–15.21, P=0.022) and chemical restraint (OR 4.67, 95% CI 1.34–16.24, p=0.015) also increased PE risk, as did benzodiazepine use (OR=3.33, 95% CI 1.34–8.30, P=0.010). Conversely, psychotropic medication before admission was associated with a lower risk of PE (OR=0.07, 95% CI 0.01–0.59, P=0.013). Stepwise multivariable forward conditional regression identified two subsets of psychiatric patients at higher risk of PE: new psychiatric cases without medication at admission who were chemically restrained, and cases without medication at admission who were started on antipsychotics and benzodiazepines. The d-dimer diagnostic tool, with an optimal threshold of 570 ng/ml determined by the Youden index (J statistic of 0.6098), showed a sensitivity of 73.17% and specificity of 87.80% for detecting PE, with an AUC of 0.833 (95% CI: 0.735–0.906).

**Conclusion:**

Our findings suggest that a history of restraint, alcoholism, and the use of antipsychotics and benzodiazepines are important predictors of PE in psychiatric inpatients. Conversely, psychotropic medications at admission may be linked to a lower PE risk. The d-dimer diagnostic tool shows good value for screening PE in psychiatric inpatients. These predictors and diagnostic markers could help clinicians identify high-risk patients and implement appropriate prevention strategies.

## 1 Introduction

Venous thromboembolism (VTE), including deep vein thrombosis (DVT) and pulmonary embolism (PE), is the world’s third-largest cardiovascular and cerebrovascular emergency (1). PE has a high intra-hospital mortality rate, and 1 in every 10 patients who died in the hospital were due to PE. Moreover, as a disease with high morbidity and mortality, PE has still shown an upward trend in recent years (2). PE is a life-threatening condition where a blood clot, usually from the deep veins of the legs, travels to the lungs and blocks blood flow (3). The pathogenesis involves thrombus formation due to venous stasis, endothelial injury, and hypercoagulability, known as Virchow’s triad (4). Clots can dislodge and obstruct the pulmonary arteries, leading to reduced oxygenation and potential right ventricular failure (3). The severity of PE can range from asymptomatic to sudden death, with treatment options varying based on the extent of the embolism and patient stability (3). Furthermore, it has been reported that 4% of unexpected sudden deaths in patients hospitalized in psychiatric departments resulted from PE (5). The common clinical manifestations of PE are shortness of breath, chest pain, hypoxia, and tachycardia, but the typical symptoms of PE, namely the triad of PE (dyspnea, chest pain, hemoptysis), are not commonly seen in clinical practice, leading to misdiagnosis from time to time, delaying the early treatment of patients with PE (6, 7). Psychiatric patients, compared with normal people, have a higher risk of DVT and PE (8), especially patients with schizophrenia and bipolar disorder (9). Patients with psychosis and bipolar have a higher rate of admission to psychiatric departments. In addition, various studies have found that the risk of PE in patients taking antipsychotics is higher than in the entire population (10, 11). There is also suggestive evidence which is conflicting that antidepressants may be associated with an increased risk of VTE (12, 13). All of these factors like psychiatric disorders, drugs, and treatment modalities may predispose patients to the development of DVT and subsequent PE. However, to our knowledge, few inpatient psychiatric facilities routinely employ antithrombotic measures and diagnostic measures for PE (14).

Furthermore, misdiagnosis and missed PE in inpatient settings are common (6, 14). Psychiatric inpatients are more prone to this vulnerability and hence it is necessary to understand the characteristics of patients presenting with PE. To the present day, systematic research on PE in psychiatric inpatients has been limited. Therefore, we conducted an age-gender-matched case-control study to investigate the exposures and predictors for PE in psychiatric inpatients, concerning diagnostic, medical, and psychiatric characteristics. In this retrospective case-control study, we aim to investigate the incidence of PE in psychiatric inpatients, focusing on the potential risk factors associated with the use of antipsychotic and antidepressant medications. Our primary hypothesis is that the administration of these psychotropic drugs increases the susceptibility of psychiatric patients to PE. Our objectives are to: 1) determine the incidence rate of PE in the studied psychiatric population; 2) identify specific risk factors, including the types of medications used and other clinical characteristics; 3) assess the impact of inpatient treatment measures on the development of PE, and 4) explore the potential of post-hospitalization test results as early indicators for PE diagnosis. Through this research, we expect to provide evidence-based recommendations for improved diagnostic and clinical management practices in psychiatric inpatient settings.

## 2 Methods

The methods used for this case-control study are described below.

### 2.1 Study design and population

**Figure 1:**
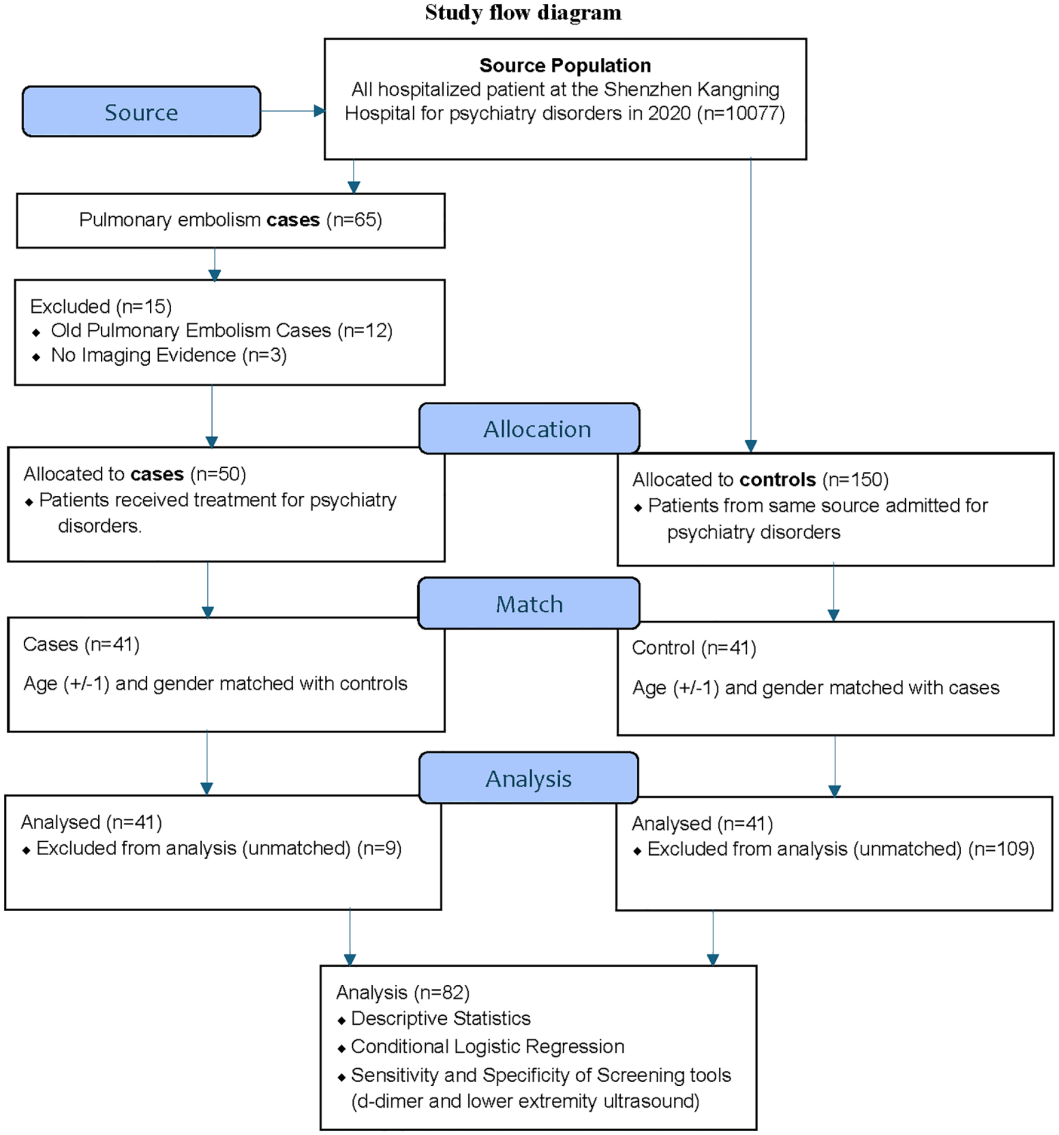
Study flow diagram.

This study is a case-control study done retrospectively to investigate the characteristics of PE in psychiatric inpatients. This retrospective study was approved by the Ethical Committee for Human Research of Shenzhen Mental Health Center, Shenzhen Kangning Hospital (2021-K015–01) and exempt from the requirement of informed consent, as it involved de-identified data acquired during the routine care of patients. **Figure 1** shows the flow diagram for the study (**Figure 1**).

### 2.2 Identification of PE cases

PE cases were identified through a two-step approach. First, all consecutive inpatients diagnosed with psychiatric disorders were included, over the year 2020, at the Shenzhen Kangning Hospital. Second, medical records were retrieved to verify the diagnosis.

The inclusion criteria for cases were:

1. Psychiatry inpatients diagnosed with PE.

The exclusion criteria for cases were:

1. Old PE cases.

2. No imaging evidence records for confirmatory diagnosis.

#### 2.2.1 Data collection and hospital record review criteria for PE diagnosis

Medical records and hospital discharge summaries were retrieved and reviewed for possible PE cases using a standardized form. The records were reviewed by two psychiatrists (DP and WZ). A diagnosis of PE was considered verified when clinical symptoms of PE supported by findings from plasma d-dimer concentration as a screening tool and contrast-enhanced computed tomography (CT) as the confirmatory tool was used. The actual imaging films were not re-interpreted. All cases with an uncertain diagnosis based on the available information were discussed until a consensus was reached. All records were checked for any notion of a previous PE event. If so, the patients were excluded. Only verified cases of new PE with imaging evidence were included as cases in the study.

### 2.3 Identification of controls

Controls were selected using a two-step approach using the survival sampling technique. Firstly, the medical records of the hospital were used to identify three controls for each case. The selected controls were psychiatric inpatients during the same year who did not have a discharge diagnosis of PE. Secondly, age and gender matching were performed, resulting in a 1:1 case-control ratio of 41. Medical records of the matched controls were retrieved and reviewed in the same manner as the medical records of the cases. Individuals who had undergone major surgery within the past three months or recently taken any medication that affects the hematopoietic system were excluded from the study.

The inclusion criteria for controls were:

1. Psychiatry inpatients not diagnosed with PE.

The exclusion criteria for cases were:

1. Major surgery within the past three months.

2. Taking any medication affecting the hematopoietic system.

### 2.4 Sample size

For our statistical calculations, we referenced a previous study on current antipsychotic use and the risk of pulmonary embolism, which provided an odds ratio (OR) of 3.68 (11). Using PASS 2021 (v21.0.3) software, we conducted a two-sided hypothesis test for the odds ratio in a matched case-control design with a binary exposure variable. Assuming an exposure probability of 0.5 and an R² of 0.05, a sample size of 39 matched sets (39 cases and 39 controls) is required to achieve a power of 0.8007 with an alpha of 0.05. However, we included all available matched cases and controls, resulting in a total of 41 matched sets. Despite the small sample size, it was justified given the low incidence and prevalence of PE in this specific population, enabling meaningful analysis within the available data.

### 2.5 Psychiatric conditions and possible confounding factors

Information regarding possible confounding factors was obtained from the medical records, using the standardized review form created using priori knowledge and literature review. Data about psychiatric conditions such as psychiatric diagnosis coded with the International Classification of Disorders 10^th^ version (ICD-10), psychotropic medications at the time of admission, drugs used after admissions (antipsychotics, antidepressants, mood stabilizers, and benzodiazepine), physical restraint (bed restraints were utilized when patients posed a threat to themselves or others), chemical restraint (injectable diazepam and/or haloperidol use) and modified electroconvulsive therapy (MECT) were obtained from medical records. The most commonly used drugs and their dose ranges in the final sample were as follows. Antipsychotics: olanzapine (2.5mg to 20mg), risperidone (1mg to 6 mg), quetiapine (25mg to 1.2g), aripiprazole (5mg to 15mg), clozapine (12.5mg to 250mg), haloperidol (5mg) and perphenazine (25mg). Antidepressants: trazodone (50mg to 100mg), fluvoxamine (50mg to 200mg), paroxetine (25mg to 40mg), sertraline (75mg to 150mg), escitalopram (5mg to 15mg), venlafaxine (75mg to 150mg), duloxetine (60mg to 120mg), mirtazapine (15mg to 30mg), and milnacipran (100mg). Mood stabilizers: lithium (0.3g to 0.9g) and valproate (0.5g to 1.5g). Benzodiazepines: lorazepam (0.5mg to 2mg), diazepam (5mg to 15mg), oxazepam (45mg), alprazolam (0.4mg to 1.2mg), clonazepam (0.5mg to 1mg), and estazolam (0.5mg to 1mg).

The other factors examined in this study extracted from the patient’s medical records included: lifestyle conditions: age, gender, alcohol use, current smoking, bedridden; and medical conditions: diabetes mellitus, dyslipidemia, hypertension, and thyroid disorder (structural and functional).

Additionally, d-dimer testing was routinely employed before inpatient psychiatry admission in the study setting, and for patients with elevated d-dimer levels, lower extremity ultrasonography was utilized as a further screening tool to detect deep vein thrombosis. Data from both tests were extracted for analysis of their sensitivity and specificity for PE.

### 2.6 Statistical methods

The contingency tables for case or control status, psychiatric conditions, and possible confounding factors were constructed. Risk estimates [odds ratios (ORs)] for possible confounding factors were estimated by conditional logistic regression. In this case-control study, with a control group matched on age and gender, we manipulated the Cox regression in Statistical Package for the Social Sciences (SPSS) to perform conditional logistic regression (15). For all analyses, a 95% Confidence Interval (CI) was estimated. In addition to univariable conditional logistic regression, a forward multivariable stepwise conditional logistic regression was also conducted using SPSS. The forward stepwise approach began with an empty model, iteratively adding variables that significantly improved model fit. Variables were included using an entry probability of 0.05 and a removal probability of 0.1, with the process continuing until no additional variables met this criterion. The best-fitted models that converged with relevant variables were reported. Data were analyzed using SPSS Version 27.

Additionally, the sensitivity and specificity of d-dimer and lower extremity ultrasonography (USG) were evaluated. Receiver Operator Characteristic (ROC) curve analysis and the optimal d-dimer value for PE screening were identified using the Youden index using MedCalc Version 22.02.

## 3 Results

The incidence of PE was 0.5% (50/10,077), and the prevalence was 0.65% (65/10,077) during the study period. The study analyzed individually matched 41 cases and 41 controls based on age and gender (**Table 1**). The median age of cases and controls was 45 (IQR 31.5–61.5) and 46 (IQR 31–61.5) years, respectively, with no significant difference between the two groups. Both groups had an equal proportion of males (n=22, 53.7%), which was ensured by the matching criteria. Rescue thrombolysis was carried out in 33 PE cases out of 41.

**Table 1:** Characteristics of age-gender matched cases and controls.

Characteristics of patients	Case, PE (n=41)	Controls (n=41)
	Median (IQR) or n (%)	
Baseline d-dimer (ng/ml)	1450 (510–3935)	310 (220–480)
USG leg (abnormal)	26 (63.4)	6 (14.6)
Length of hospital stay (days)	13 (4–29.50)	28 (18.5–38.5)
Age (years)	45 (31.5–61.5)	46 (31–61.5)
Male	22 (53.7)	22 (53.7)
Active smoker (Yes)	14 (34.1)	10 (24.4)
Active alcoholism (Yes)	16 (39)	8 (19.5)
Bedridden (Yes)	3 (7.3)	2 (4.9)
Diabetes mellitus (Yes)	5 (12.2)	6 (14.6)
Hypertension (Yes)	14 (34.1)	7 (17.1)
Dyslipidemia (Yes)	4 (9.8)	5 (12.2)
Thyroid disorder (Yes)	6 (14.6)	5 (12.2)
ICD 10 diagnosis		
F0-F1	10 (24.4)	6 (14.6)
F2	12 (29.3)	12 (29.3)
F3-F4	16 (39)	20 (48.8)
Others	3 (7.3)	3 (7.3)
On psychotropics (Yes)	26 (63.4)	38 (92.7)
Current antipsychotic (Yes)	38 (92.7)	33 (80.5)
First-generation antipsychotic (Yes)	10 (24.4)	3 (7.3)
Current antidepressants (Yes)	10 (24.4)	12 (29.3)
Current mood stabilizer (Yes)	14 (34.1)	15 (36.6)
Current benzodiazepines (Yes)	24 (58.5)	10 (24.4)
History of physical restraint (Yes)	16 (39)	6 (14.6)
History of chemical restraint (Yes)	14 (34.1)	3 (7.3)
History of MECT (Yes)	10 (31)	4 (9.8)

PE, pulmonary embolism; n, frequency; IQR, Interquartile range; ICD, International Classification of Disorder; F0, organic, including symptomatic, mental disorders; F1, mental and behavioral disorders due to psychoactive substance use; F2, schizophrenia, schizotypal and delusional disorders; F3, mood [affective] disorders (e.g., major depressive disorder, bipolar disorder); F4, neurotic, stress-related, and somatoform disorders (e.g., anxiety disorders, PTSD); MECT, modified electroconvulsive disorder; USG leg, ultrasound for lower extremity; On psychotropics, on psychotropic medications at admission.

### 3.1 Characteristics of age-gender matched cases and controls

#### 3.1.1 Medical and living conditions

The median length of hospital stay was shorter in cases compared to controls (13 days vs. 28 days). A higher proportion of cases had a history of hypertension compared to controls (34.1% vs. 17.1%). A higher proportion of cases reported active smoking (34.1% vs 24.4%), and active alcoholism (39% vs 19.5%) compared to controls. The proportions of cases who were bedridden (7.3% vs 4.9%), had diabetes mellitus (12.2% vs 14.6%), dyslipidemia (9.8% vs 12.2%), and thyroid disorders (14.6% vs 12.2%) was similar to that of controls.

#### 3.1.2 Psychiatric conditions

A significantly lower proportion of cases were on psychotropic medications at admission compared to controls (63.4% vs. 92.7%). Among cases, a higher proportion was observed currently taking benzodiazepines compared to controls (58.5% vs. 24.4%) and on antipsychotic medication (92.7% vs 80.5%). The proportion of patients currently on antidepressants (24.4% vs 29.3%) and mood stabilizers (34.1% vs 36.6%) was similar in cases and controls. A higher proportion of cases had a history of physical restraint (39% vs. 14.6%), chemical restraint (34.1% vs 7.3%), and MECT (31% vs. 9.8%) compared to controls.

### 3.2 Conditional logistic regression analysis of predictors of PE

**Table 2** shows the results of an univariable conditional logistic regression analysis of predictors of PE (**Table 2**). The OR with 95% CI and p-values are presented for each predictor variable. The variables that were found to be statistically significant predictors of PE were: active alcoholism (OR 3.67, 95% CI 1.02–13.14, p=0.046), patient under psychotropic medication at admission (OR 0.07, 95% CI 0.01–0.59, p=0.013), current benzodiazepines use (OR 3.33, 95% CI 1.34–8.30, p=0.010), history of physical restraint (OR 4.33, 95% CI 1.24–15.21, p=0.022) and history of chemical restraint (OR 4.67, 95% CI 1.34–16.24, p=0.015).

**Table 2:** Univariable conditional logistic regression analysis of predictors of PE.

Variables	OR (95% CI)	P value
Length of hospital stay (days)	0.97 (0.948–1.00)	0.050
Active smoker (Yes)	2.33 (0.60–9.02)	0.220
Active alcoholism (Yes)	3.67 (1.02–13.14)	0.046
Bedridden (Yes)	1.50 (0.25–8.98)	0.657
Diabetes mellitus (Yes)	0.50 (0.05–5.51)	0.571
Hypertension (Yes)	4.50 (0.97–20.83)	0.054
Dyslipidemia (Yes)	0.67 (0.11–3.99)	0.657
Thyroid disorder (Yes)	1.25 (0.37–4.65)	0.739
ICD 10 diagnosis		
F0-F1	Reference	0.741
F2	0.67 (0.15–2.99)	0.595
F3-F4	0.51 (0.16–1.65)	0.263
Others	0.69 (0.08–6.13)	0.739
On psychotropics (Yes)	0.07 (0.01–0.59)	0.013
Current antipsychotic (Yes)	3.50 (0.73–16.85)	0.118
First-generation antipsychotic (Yes)	3.33 (0.92–12.11)	0.067
Current antidepressants (Yes)	0.80 (0.32–2.03)	0.638
Current mood stabilizer (Yes)	0.86 (0.29–2.55)	0.782
Current benzodiazepines (Yes)	3.33 (1.34–8.30)	0.010
History of physical restraint (Yes)	4.33 (1.24–15.21)	0.022
History of chemical Restraint (Yes)	4.67 (1.34–16.24)	0.015
History of MECT (Yes)	3.00 (0.81–11.08)	0.099

PE, pulmonary embolism; n, frequency; IQR, Interquartile range; ICD, International Classification of Disorder; F0, organic, including symptomatic, mental disorders; F1, mental and behavioral disorders due to psychoactive substance use; F2, schizophrenia, schizotypal and delusional disorders; F3, mood [affective] disorders (e.g., major depressive disorder, bipolar disorder); F4, neurotic, stress-related, and somatoform disorders (e.g., anxiety disorders, PTSD); MECT, modified electroconvulsive disorder; USG leg, ultrasound for lower extremity; On psychotropics, on psychotropic medications at admission; OR, odds ratio; CI, confidence interval.

Other variables that were not found to be statistically significant predictors of PE during univariable analysis include the length of hospital stay, smoking, diabetes mellitus, hypertension, dyslipidemia, thyroid disorder, ICD 10 Classification of psychiatric diagnosis, current antipsychotic use, current antidepressants use, current mood stabilizer use, and history of MECT.

The forward stepwise multivariable conditional logistic regression analysis was carried out iteratively using an entry probability of 0.05 and a removal probability of 0.1 which identified two subsets of the model as valid and fitted. The first subset included three variables with significance: medication before admission [Adjusted OR (AOR) 0.09, 95% CI 0.01–0.76, p=0.027], current benzodiazepine use (AOR 3.70, 95% CI 1.16–11.81, p=0.028), and antipsychotic use (AOR 8.66, 95% CI 1.05–71.57, p=0.045). The second subset included two variables with significance: medication before admission (AOR 0.86, 95% CI 0.11–0.702, p=0.022) and history of chemical restraint (AOR 4.09, 95% CI 1.03–16.28, p=0.045).

### 3.3 Screening tools

**Figure 2:**
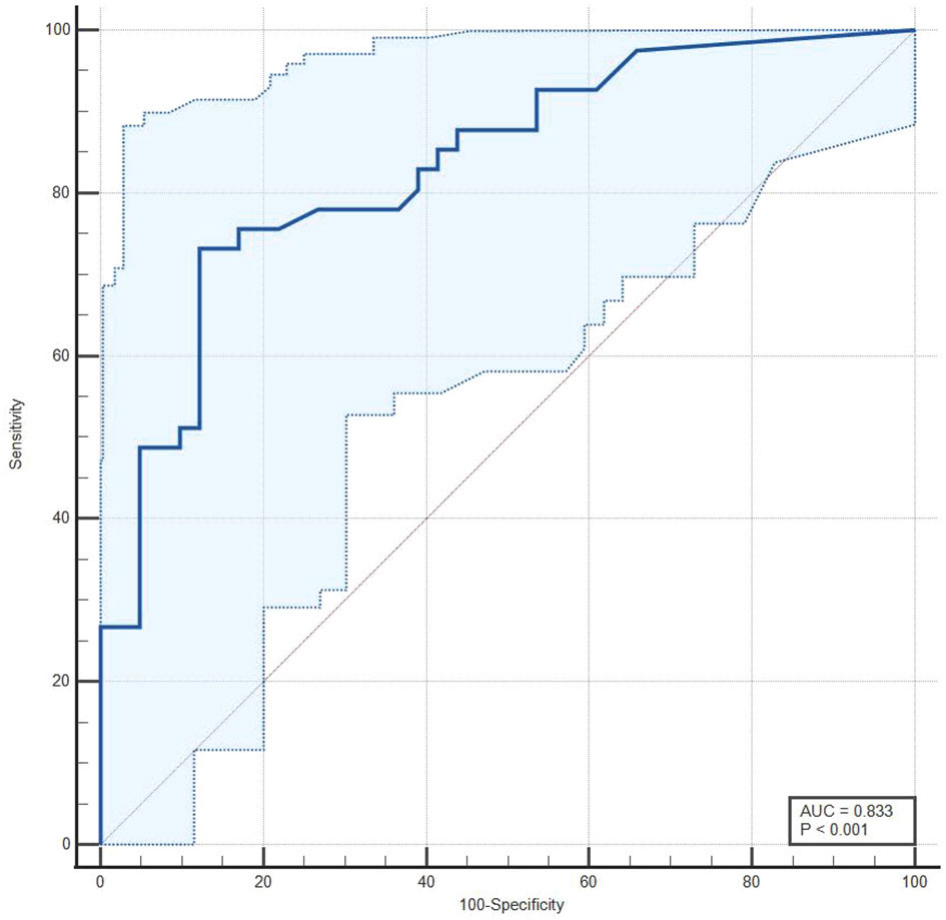
A receiver operating characteristic (ROC) curve for d-dimer test in the diagnosis of PE in psychiatric inpatients.

The median baseline d-dimer level was higher in cases with PE compared to controls (1450 ng/mL vs. 310 ng/mL). A higher proportion of cases had an abnormal ultrasound for the lower extremity compared to controls (63.4% vs. 14.6%). **Table 3** presents the sensitivity and specificity of screening tools for the diagnosis of PE (**Table 3**). For baseline d-dimer levels greater than 570 ng/ml, the sensitivity was 73.17% and the specificity was 87.80%. When using ultrasound for the lower extremities (USG Leg) with a positive DVT result, the sensitivity for PE diagnosis was 63.41% and the specificity was 85.36%. The optimal threshold for d-dimer levels was identified as 570 ng/ml through the application of the Youden index, which yielded a J statistic of 0.6098. **Figure 2** illustrates the receiver operating characteristic (ROC) curve for the d-dimer test in diagnosing PE among psychiatric inpatients (**Figure 2**). The area under the curve (AUC), with a value of 0.833 and a 95% CI ranging from 0.735 to 0.906, indicates the test’s strong potential for detecting PE in a psychiatric inpatient facility.

**Table 3:** Sensitivity and specificity of screening tools for PE diagnosis.

Variables	Value	Sensitivity	Specificity
Baseline D-dimer (ng/ml)	>570 ng/ml*	73.17	87.80
USG leg	DVT Positive	63.41	85.36

PE, pulmonary embolism; USG leg, ultrasound for lower extremity; DVT, deep venous thrombosis. *Youden index (J = 0.6098).

## 4 Discussion

The study found that the incidence of PE in a psychiatric inpatient facility was 0.5%, with a prevalence of 0.65%. Univariable conditional logistic regression analysis revealed that patients with active alcoholism, a history of restraint, and current benzodiazepine use had a higher risk of developing PE. Conversely, patients who were on psychotropic medication before admission showed a lower risk of PE. Further analysis using multivariable stepwise forward conditional regression identified that current use of antipsychotics and benzodiazepines increased the risk of PE, while prior use of psychotropic medications before admission continued to be associated with a reduced risk of PE.

According to our findings, the incidence of PE in psychiatry inpatients was 0.5%, which is consistent with previous studies. Another study reported an incidence of 0.67% (3/449) of PE in hospitalized psychiatric patients after 90 days of follow-up (16), while a study of patients without surgery in other departments found that 0.119% developed PE during hospitalization (17).

In the univariable conditional logistic regression analysis, alcohol consumption was significantly associated with an increased risk of PE. However, this significance did not hold in the multivariable stepwise conditional regression analysis, likely due to the presence of confounding variables that, when accounted for, diminish the apparent effect of alcohol consumption on PE risk. The study’s finding of a significant association between alcohol consumption and increased PE risk contradicts previous research that found no such significant association (18, 19). The authors suggest that the observed differences may be due to the synergistic effects of alcohol use and psychiatric risk factors, which together may increase the likelihood of developing PE. It is well known that smoking increases the risk of VTE (20). Even though there were more smokers in the PE group, the current study didn’t find any statistically significant association between smokers and PE which is consistent with the population-based study done in China (21). The study also identified no significant differences between cases and controls regarding comorbidities such as diabetes mellitus, hypertension, dyslipidemia, and thyroid disorder, aligning with other studies on psychiatric inpatients (22).

In contrast to previous research, our study revealed that psychiatric inpatients who were on psychotropic medications upon admission had a lower chance of having PE (10–13). Moreover, our study found no association between current antidepressants, and mood stabilizers and the risk of PE. However stepwise conditional regression found current antipsychotics use have high risk of PE. This finding contradicts previous studies, which suggested that antidepressant medications have increased the risk of VTE (12, 13) but is in line with studies that suggest that the use of antipsychotics has increased the risk of VTE (10, 11). The patients without psychotropic medications are new cases admitted in psychiatry infacility, authors speculate PE might have been misdiagnosed and initially treated as psychiatric cases. It is important to note, however, that this study specifically focused on psychiatric inpatients, who may have different risk factors for PE compared to other patient populations that is why the results also showed that cases had a shorter median hospital stay compared to controls, which is unexpected as PE is usually associated with a longer hospital stay because PE cases were transferred from the psychiatry department to other departments after diagnosis. Nonetheless, our study findings are consistent with prior research that identified the use of benzodiazepines and restraint as significant predictors of PE, both chemical and mechanical restraint were significant (23, 24). However, the use of benzodiazepines and chemical restraint holds significance in the subsets of multivariable stepwise conditional regression analysis but the history of physical restraint did not.

The analysis of screening tools revealed that the cases had a higher median baseline d-dimer level than the controls, implying a potential connection between elevated d-dimer levels and PE. This finding is in line with the current guidelines for the detection and management of PE, which suggest using d-dimer levels as a diagnostic tool (25). The study found that baseline d-dimer levels greater than 570 ng/ml, have a sensitivity of 73.17% and a specificity of 87.80%. Moreover, a higher proportion of the cases demonstrated an abnormal finding on the lower extremity ultrasound, which is in line with previous investigations linking DVT to PE (5). Nevertheless, the research also highlights that almost half of the VTE patients (19 out of 39) developed PE without having DVT. For lower limb venous ultrasound in this study sensitivity for PE diagnosis was 63.41% and the specificity was 85.36%. Since PE cannot be detected through a lower limb venous ultrasound, it could be missed unless a contrast-enhanced CT scan is performed (5). Therefore, a cautious application of d-dimer and lower limb venous ultrasound in combination with clinical features could facilitate the early detection of PE in psychiatric settings.

The study identified some factors that may increase the risk of pulmonary embolism in psychiatric inpatients, such as alcohol consumption, antipsychotic use, benzodiazepine use, and history of the restraint, and highlighted the importance of d-dimer and lower extremity ultrasound in the detection of pulmonary embolism. Serum d-dimer detection and lower extremity ultrasound are cost-effective and convenient as monitoring index or screening tools for PE before confirming tools like pulmonary angiography CT. Furthermore, new cases without psychotropic medication should warrant full physical checkups for potentially life-threatening conditions.

## 5 Clinical implication and future direction

In our settings, a d-dimer test is performed before admission. Additionally, regular VTE training is conducted to enhance capacity building. This approach helps in the early identification, treatment, and prevention of mortality due to PE.

As per treatment protocol at Shenzhen Mental Health Center, Shenzhen Kangning Hospital, when the d-dimer level is between 500–1000 ng/mL, a lower extremity ultrasound is conducted to exclude DVT. If the d-dimer continues to rise above 1000 ng/mL, a CTA (computed tomography angiography) is performed to exclude PE. If PE is confirmed, it is recommended to consult a general hospital or transfer the patient for specialized treatment. In some cases, anticoagulation therapy with rivaroxaban or low molecular weight heparin is initiated.

Clinicians consider multiple factors such as patient age, restraints, and costs when deciding whether to perform pulmonary angiography. It’s crucial to be vigilant about the possibility of PE in psychiatric patients, especially those exhibiting hyperarousal symptoms like shortness of breath, chest pain, and tachycardia, even if they are not on psychotropic medications. Routine d-dimer testing upon admission can aid in the early diagnosis and prevention of PE-related deaths, despite the triad of PE symptoms (dyspnea, chest pain, hemoptysis) being uncommon in clinical practice.

Our study identified two subsets of psychiatric patients at risk of PE: 1) new psychiatric cases without medication at admission who were chemically restrained and 2) cases without medication at admission who were initiated on antipsychotics and benzodiazepines.

The implementation of early diagnostic protocols and regular capacity building for PE in our psychiatric facility has significantly contributed to reducing mortality rates associated with PE.

## 6 Limitation

The study was retrospective, which means that the researchers could only analyze data that was already available. This limits the types of analyses that can be performed and introduces the possibility of missing or incomplete data. The sample size was relatively small and conducted at a single medical center, which limits the generalizability of the findings. A larger sample size would be needed to confirm the results and generalize them to other populations. The study only looked at patients with a confirmed diagnosis of PE, which may not be representative of all patients with suspected PE. The study did not collect data on other potential risk factors for PE, such as family history, surgery, or recent travel and duration/dose of drug use. This limits the ability to fully understand the risk factors for PE in this population.

## 7 Conclusion

The study investigated the prevalence and potential risk factors in psychiatric inpatients as well as diagnostic tests for PE. Our findings suggest that a combination of diagnostic, medical, and psychiatric factors should be taken into account when diagnosing PE in this patient population. Specifically, our study revealed that current benzodiazepine use and antipsychotic use were significantly associated with an increased risk of PE, while patients on psychotropic medications upon admission had a lower chance of having PE. We also confirmed the importance of using d-dimer and lower extremity vascular Doppler ultrasound to check for PE.

Further research is needed to confirm these findings and identify potential mechanisms underlying the observed associations. Nonetheless, these findings provide important insights into the factors that may affect the occurrence of PE in patients in psychiatric hospitals and could help guide future clinical practice in this population.

## Data Availability

The raw data supporting the conclusions of this article will be made available by the authors, without undue reservation.

## References

[1] Konstantinides SV Meyer G. The 2019 ESC guidelines on the diagnosis and management of acute pulmonary embolism. Eur Heart J. (2019) 40:3453–5. doi: 10.1093/eurheartj/ehz726 31697840

[2] Dentali F Ageno W Pomero F Fenoglio L Squizzato A Bonzini M. Time trends and case fatality rate of in-hospital treated pulmonary embolism during 11 years of observation in Northwestern Italy. Thromb Haemost. (2016) 115:399–405. doi: 10.1160/th15-02-0172 26422774

[3] Turetz M Sideris AT Friedman OA Triphathi N Horowitz JM. Epidemiology, pathophysiology, and natural history of pulmonary embolism. Semin Interv Radiol. (2018) 35:92–8. doi: 10.1055/s-0038-1642036 PMC5986574 29872243

[4] Stone J Hangge P Albadawi H Wallace A Shamoun F Knuttien MG. Deep vein thrombosis: pathogenesis, diagnosis, and medical management. Cardiovasc Diagn Ther. (2017) 7(Suppl 3):S276–S84. doi: 10.21037/cdt.2017.09.01 PMC5778510 29399531

[5] Takeshima M Ishikawa H Shimizu K Kanbayashi T Shimizu T. Incidence of venous thromboembolism in psychiatric inpatients: a chart review. Neuropsychiatr Dis Treat. (2018) 14:1363–70. doi: 10.2147/NDT PMC5973315 29872303

[6] Kwok CS Wong CW Lovatt S Myint PK Loke YK. Misdiagnosis of pulmonary embolism and missed pulmonary embolism: A systematic review of the literature. Health Sci Review. (2022) 3:100022. doi: 10.1016/j.hsr.2022.100022

[7] Morrone D Morrone V. Acute pulmonary embolism: focus on the clinical picture. Korean Circ J. (2018) 48:365–81. doi: 10.4070/kcj.2017.0314 PMC5940642 29737640

[8] Thomassen R Vandenbroucke JP Rosendaal FR. Antipsychotic medication and venous thrombosis. Br J Psychiatry. (2001) 179:63–6. doi: 10.1192/bjp.179.1.63 11435271

[9] Strudsholm U Johannessen L Foldager L Munk-Jørgensen P. Increased risk for pulmonary embolism in patients with bipolar disorder. Bipol Disord. (2005) 7:77–81. doi: 10.1111/j.1399-5618.2004.00176.x 15654935

[10] Allenet B Schmidlin S Genty C Bosson JL. Antipsychotic drugs and risk of pulmonary embolism. Pharmacoepidemiol Drug Saf. (2012) 21:42–8. doi: 10.1002/pds.2210 22052683

[11] Liu Y Xu J Fang K Xu Y Gao J Zhou C. Current antipsychotic agent use and risk of venous thromboembolism and pulmonary embolism: a systematic review and meta-analysis of observational studies. Ther Adv Psychopharmacol. (2021) 11:2045125320982720. doi: 10.1177/2045125320982720 33505665 PMC7812411

[12] Wang Y Ye Z Liu L Cui X. Antidepressant use and risk of venous thromboembolism: A systematic review and meta-analysis. J Pharm Pharm Sci. (2019) 22:57–71. doi: 10.18433/jpps30129 30660205

[13] Kunutsor SK Seidu S Khunti K. Depression, antidepressant use, and risk of venous thromboembolism: systematic review and meta-analysis of published observational evidence. Ann Med. (2018) 50:529–37. doi: 10.1080/07853890.2018.1500703 30001640

[14] Amy Barnhorst MD Glen L. Xiong MD. Pulmonary embolism in a psychiatric patient. Am J Psychiatry. (2014) 171:1155–7. doi: 10.1176/appi.ajp.2013.13040494 25756631

[15] IBM Support. Conditional logistic regression using COXREG (2020). Available online at: https://www.ibm.com/support/pages/conditional-logistic-regression-using-coxreg.

[16] Delluc A Montavon S Canceil O Carpentier M Nowak E Mercier B. Incidence of venous thromboembolism in psychiatric units. Thromb Res. (2012) 130:e283–8. doi: 10.1016/j.thromres.2012.10.002 23092750

[17] Imura M Yamamoto T Hiasa K-I. Pulmonary thromboembolism developed during hospitalization: A nationwide retrospective observational study using claims data. Cardiol Ther. (2023) 12:127–41. doi: 10.1007/s40119-022-00290-6 PMC9734681 36482141

[18] Chen M Ji M Chen T Hong X Jia Y. Alcohol consumption and risk for venous thromboembolism: A meta-analysis of prospective studies. Front Nutr. (2020) 7. doi: 10.3389/fnut.2020.00032 PMC7145405 32300598

[19] Harrington LB Hagan KA Mukamal KJ Kang JH Kim J Crous-Bou M. Alcohol consumption and the risk of incident pulmonary embolism in US women and men. J Thromb Haemost. (2018) 16:1753–62. doi: 10.1111/jth.14224 PMC6368406 29974610

[20] Cheng YJ Liu ZH Yao FJ Zeng WT Zheng DD Dong YG. Current and former smoking and risk for venous thromboembolism: a systematic review and meta-analysis. PloS Med. (2013) 10:e1001515. doi: 10.1371/journal.pmed.1001515 24068896 PMC3775725

[21] Chan KH Wright N Xiao D Guo Y Chen Y Du H. Tobacco smoking and risks of more than 470 diseases in China: a prospective cohort study. Lancet Public Health. (2022) 7:e1014–e26. doi: 10.1016/S2468-2667(22)00227-4 PMC7613927 36462513

[22] Takeshima M Ishikawa H Umeta Y Kudoh M Umakoshi A Yoshizawa K. Prevalence of asymptomatic venous thromboembolism in depressive inpatients. Neuropsychiatr Dis Treat. (2020) 16:579–87. doi: 10.2147/NDT PMC7049756 32161463

[23] Chen T-Y Winkelman JW Mao W-C Tzeng N-S Kuo TBJ Yang CCH. Real-world evidence on the use of benzodiazepine receptor agonists and the risk of venous thromboembolism. J Thromb Haemostasis. (2020) 18:2878–88. doi: 10.1111/jth.15033 32741123

[24] Hirose N Morita K Nakamura M Fushimi K Yasunaga H. Association between the duration of physical restraint and pulmonary embolism in psychiatric patients: A nested case–control study using a Japanese nationwide database. Arch Psychiatr Nursing. (2021) 35:534–40. doi: 10.1016/j.apnu.2021.07.009 34561070

[25] Konstantinides SV Meyer G Becattini C Bueno H Geersing GJ Harjola VP. 2019 ESC Guidelines for the diagnosis and management of acute pulmonary embolism developed in collaboration with the European Respiratory Society (ERS). Eur Heart J. (2020) 41:543–603. doi: 10.1093/eurheartj/ehz405 31504429

